# Holocene polynya dynamics and their interaction with oceanic heat transport in northernmost Baffin Bay

**DOI:** 10.1038/s41598-021-88517-9

**Published:** 2021-05-12

**Authors:** Rebecca Jackson, Anna Bang Kvorning, Audrey Limoges, Eleanor Georgiadis, Steffen M. Olsen, Petra Tallberg, Thorbjørn J. Andersen, Naja Mikkelsen, Jacques Giraudeau, Guillaume Massé, Lukas Wacker, Sofia Ribeiro

**Affiliations:** 1 Department of Glaciology and Climate, Geological Survey of Denmark and Greenland, Øster Voldgade 10, 1350 Copenhagen, Denmark; 2 Department of Geosciences and Natural Resource Management, University of Copenhagen, Øster Voldgade 10, 1350 Copenhagen, Denmark; 3 Department of Earth Sciences, University of New Brunswick, 2 Bailey Drive, Fredericton, E3B 5A3 Canada; 4 Department of Geography, Durham University, Lower Mountjoy, South Road, DH1 3LE Durham, UK; 5 Université Bordeaux, CNRS, EPHE, UMR 5805 EPOC, Allée Geoffroy Saint-Hilaire CS 50023, 33615 Pessac Cedex, France; 6 Danish Meteorological Institute (DMI), Lyngbyvej 100, 2100 Copenhagen, Denmark; 7 Faculty of Biological and Environmental Sciences, University of Helsinki, P.O. Box 65, Helsinki, 00014 Finland; 8 Station Marine De Concarneau, Place De La Croix, 29900 Concarneau, France; 9 Université Laval, CNRS, UM 3376 TAKUVIK, Allée de la Médecine, Québec, G1V0A6 Canada; 10 Ion Beam Physics, Physics, Swiss Federal Institute of Technology in Zurich, Otto-Stern-Weg 5, 8093 Zürich, Switzerland

**Keywords:** Palaeoceanography, Palaeoclimate

## Abstract

Baffin Bay hosts the largest and most productive of the Arctic polynyas: the North Water (NOW). Despite its significance and active role in water mass formation, the history of the NOW beyond the observational era remains poorly known. We reconcile the previously unassessed relationship between long-term NOW dynamics and ocean conditions by applying a multiproxy approach to two marine sediment cores from the region that, together, span the Holocene. Declining influence of Atlantic Water in the NOW is coeval with regional records that indicate the inception of a strong and recurrent polynya from ~ 4400 yrs BP, in line with Neoglacial cooling. During warmer Holocene intervals such as the Roman Warm Period, a weaker NOW is evident, and its reduced capacity to influence bottom ocean conditions facilitated northward penetration of Atlantic Water. Future warming in the Arctic may have negative consequences for this vital biological oasis, with the potential knock-on effect of warm water penetration further north and intensified melt of the marine-terminating glaciers that flank the coast of northwest Greenland.

## Introduction

Despite being perceived as a cold and barren environment, the marine Arctic sustains oases of biological productivity, connected to the prominent winter sea-ice polynyas. These areas of open water in the otherwise sea-ice covered high latitude environments serve as feeding grounds and refuge to a plethora of marine mammals and birds^1^. Today, the North Water Polynya (NOW) is the largest and most biologically productive coastal polynya in the Arctic and is of significant cultural and historical importance to Inuit communities in Greenland and Canada^2^. The NOW affects the regional climate and ocean circulation by being an active moisture and winter heat source and via deep winter mixing associated with brine release from intense sea ice production^3,4^. Deepwater formation in the NOW is thought to contribute to the Baffin Bay Bottom Water mass^5^ and has been suggested as a potential source of a newly described current flowing southward along the banks of West Greenland^4,6^.

**Figure 1:**
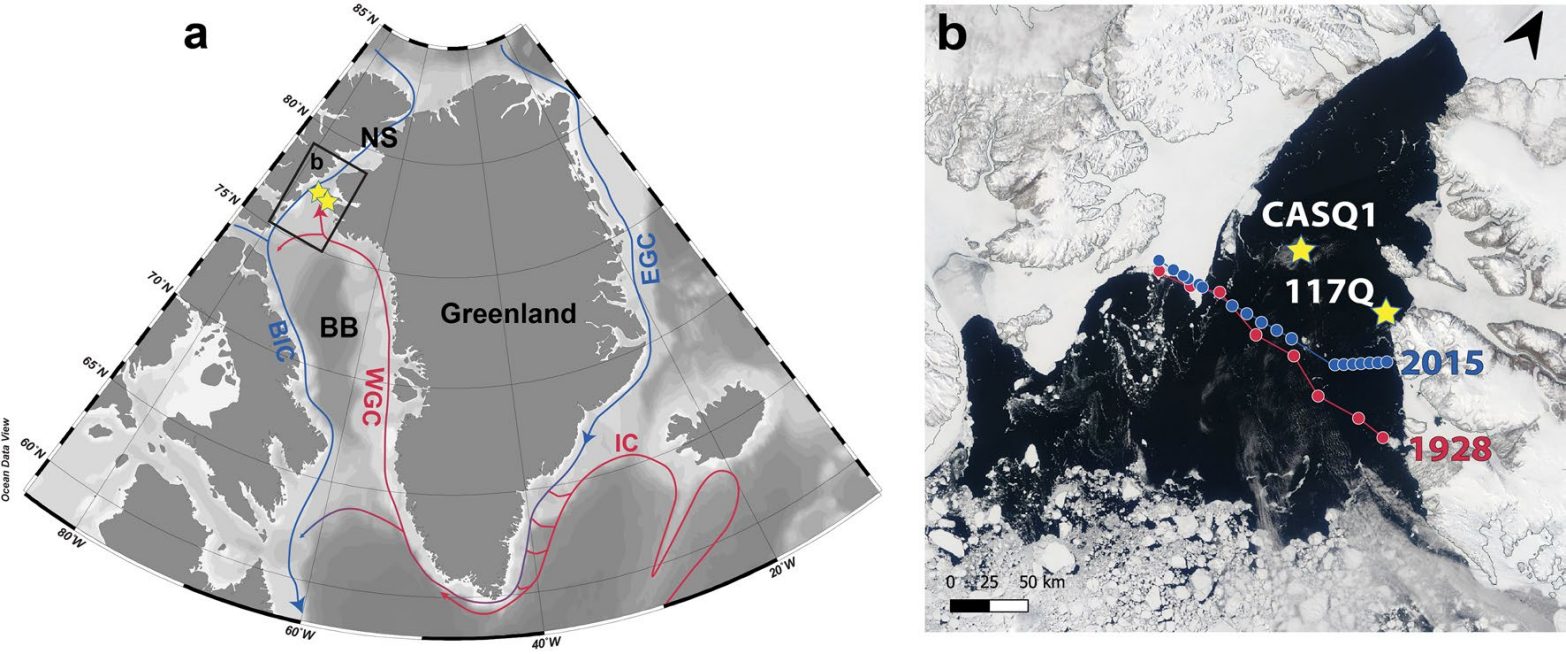
Study area and satellite imagery of the North Water Polynya. (**a**) Geographical location of the North Water Polynya (NOW) and sediment cores (yellow stars) in northernmost Baffin Bay (BB) south of Nares Strait (NS). Regional warm (red) and cold (blue) ocean currents include *BIC* Baffin Island Current, *WGC* West Greenland Current, *IC* Irminger Current, *EGC* East Greenland Current. Insert (**b**) Satellite image (MODIS; NASA worldview) of the NOW region on the 26 May 2008 and central (CASQ1) and peripheral (117Q) core sites and the location of the CTD transects from 1928 (red) and 2015 (blue) in Fig. 2.

The NOW recurrently forms in northern Baffin Bay from March and remains open until October. Despite interannual variability, the polynya extends spatially to around 80,000 km^2^. Primary production in the open waters of the NOW is exceptionally high for a polar marine environment (e.g.^7^) with surface sedimentary organic carbon concentrations of up to 2%^8^. Diatoms are the main driver of primary production and the NOW is an effective biogenic silica sink; fluxes can exceed 25 mmol Si m^−2^ d^−1^^9^ and subsequent silica dissolution is the cause of the silicate anomaly in deep Baffin Bay^10^. Fundamental to the physical configuration of the NOW is the formation in the winter period of an ice-arch in Smith Sound/Kane Basin, seasonally blocking Arctic sea ice export to northern Baffin Bay^3,11^ (Fig. 1). Coupled with the ice-arch, the dominant northerly winds and ocean circulation ensure effective removal of newly formed sea ice, thus defining it essentially as a latent heat polynya^4,12^.

Satellite data highlight the spatial variability of the NOW over recent decades. In 2007, for example, the ice-arch in Smith Sound failed to form^13^. Recent analysis of the last four decades of satellite data indicate ice-arch duration decreased to 128 days/year from a pre-2007 average of 177 days/year^14^ and over the last 20 years, decreasing ice-arch duration has been concomitant with increasing ice area and ice export through Nares Strait^15^. Further back in time, lake sediment records^16^ point to the inception of a stable and productive NOW from ~4400 yrs BP, evident from the first arrival of little auk, a seabird species that depends on the early open water of the polynya for feeding. Marine records from Kane Basin (Nares Strait), to the north of the NOW, indicate harsh sea-ice conditions favourable for ice-arch formation from 5000–3500 yrs BP, likely prompted by cooler atmospheric temperatures and predominantly negative phases of the Arctic Oscillation^17^.

**Figure 2:**
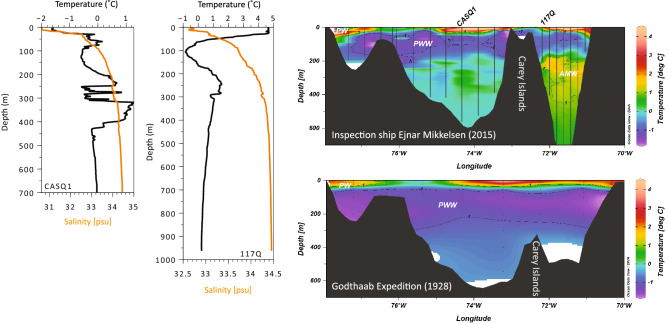
Water column characteristics in the NOW region. Temperature and salinity profiles (left) at the core locations of CASQ1 (08.10.2015) and 117Q (07.08.2016). Temperature profiles from CTD transects taken during the Ejnar Mikkelsen expedition (2015, top right) and bottle samples from the Godthaab Expedition^21^ (1928; bottom right, data from http://www.ices.dk). Black vertical lines/dots indicate CTD station/bottle positions. *PW* Polar Water, *PWW* Polynya Winter Water, *AMW* Atlantic Mode Water. For location of transects see Fig. 1b.

Below the surface waters, the NOW is connected to the Atlantic via the northward flowing West Greenland Current (WGC). Evidence of the WGC, a combination of the cold ice-loaded East Greenland Current (EGC) and Atlantic-sourced Irminger Current (IC) (Fig. 1), has been observed as far north as Smith Sound^17–19^ and Inglefield Bredning^20^. Today (Fig. 2), the Atlantic-sourced layer has a core depth of 300 m and is associated with temperatures exceeding 1 °C. Winter waters near ocean freezing temperature and, modified or locally produced by polynya processes, occupy the water column above, shoaling towards the east. Other circulation regimes have existed though time; comparing observed conditions from 1928 (Godthaab Expedition^21^) with today’s hydrography for example clearly illustrates an absence of Atlantic-sourced water (Atlantic Mode Water, AMW, also referred to as Atlantic Intermediate Water, Fig. 2) and indications of vigorous, deep winter mixing as seen by a deep layer of Polynya Winter Water (PWW) in 1928 (Fig. 2).

Multi-proxy studies from the Nordic Seas highlighted the vital role of coastal polynyas in sustaining deep water formation during the Last Glacial Maximum^22^. The influence of corrosive (CO_2_-rich, low pH) brines produced by these ‘sea-ice factories’^23^ extends throughout the water column; higher proportions of agglutinated (vs. calcareous) benthic foraminifera characterise periods of polynya stability in the fjords of Svalbard^24–26^. A shift toward polar, predominantly agglutinated, assemblages at ~ 4000 yrs BP in the western sector of the modern-day NOW was attributed to increasingly polynya-like conditions^27^. From the few marine records that capture early Holocene timescales in the NOW, benthic foraminifera indicate maximum influx of Atlantic water via the WGC between ~ 10,800–8200 yrs BP^28^. The impact of major ice sheet reconfigurations, culminating in the opening of the Nares Strait between 9000 and 8300 yrs BP^18,19,29^ (see^27^ for alternative timing), on ocean circulation and polynya formation in northern Baffin Bay is yet to be explored.

A fully comprehensive study of the history of the NOW requires not only paleo- reconstructions of sea-surface conditions and ocean circulation, but consideration of the NOW itself as an influencing entity, interacting with and initiating change in the marine system. Here, we reconcile the formation and dynamics of the NOW and its interactions with bottom ocean conditions in northernmost Baffin Bay. Using two well-dated marine sediment archives from contrasting regions of the modern-day NOW, we apply a suite of proxies charting sea-ice conditions (lipid biomarkers), surface productivity (total organic carbon and biogenic silica) and bottom water characteristics (benthic foraminifera assemblages). Reconstructions based on marine sediments from the central NOW, covering the late Holocene (CASQ1, Fig. 1b) are compared with a full Holocene record from it's  eastern periphery (117Q, Fig. 1b). The location of 117Q is advantageous; it lies directly below the sea ice margin of the modern-day NOW and the main pathway of Atlantic-sourced waters into northernmost Baffin Bay.

## Results

### Geochronology

**Figure 3:**
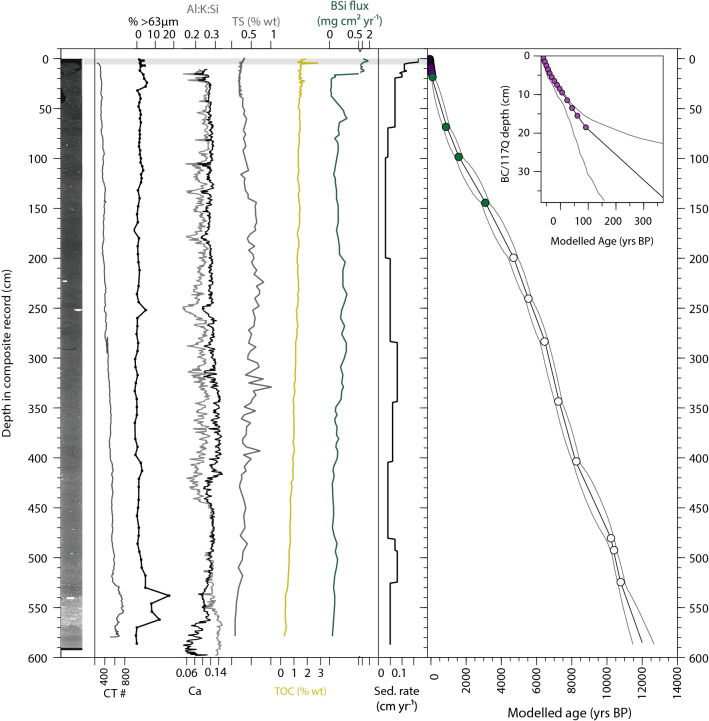
Physical and selected biogeochemical sediment properties and age-depth model for 117Q BC and 117Q. From left to right: 117Q Computerised tomography (CT) scan image and CT number (#). Denser areas appear whiter in the CT scan image. The sand fraction (% > 63 µm) measured by wet sieving (black). Variations in XRF-measured elements calcium (Ca; black) and sum of Al, K and Si (grey), both are shown as ratio to the sum of total elemental counts. Percentage (weight) of total sulphur (TS; grey) and total organic carbon (TOC; yellow) and biogenic silica fluxes (BSi; dark green). Calculated sedimentation rates (cm yr^-1^) are shown in black. The median age (modelled) -depth relationship constructed in BACON for 117Q and 117Q BC (insert) are shown with purple-filled circles representing ^210^Pb-dated intervals, green-filled circles indicating the bulk organic carbon ^14^C-dated intervals and open circles the ^14^C-dated intervals (biogenic carbonate). The solid black line indicates the median modelled age-depth relationship and the grey dashed lines indicate maximum and minimum modelled ages in the 95% confidence interval. The grey bar indicates the stratigraphic interval where 117Q BC and 117Q proxy data overlap.

A combined ^210^Pb (n = 14) and ^14^C (n = 17) chronology of 117Q and the 117Q box core (BC) indicates that the whole core record covers the period 12,000 yrs BP (± 600 yrs) to − 62 yrs BP (Fig. 3). ^210^Pb dating on the top of 117Q and 117Q BC sediments indicate an overlap of 6.5 cm (Supplementary Fig. 1). Three replicate ^14^C dates from the same 1 cm interval (524.5 cm) in 117Q measured on planktonic foraminifera, mixed benthic foraminifera and bivalve shells reported ^14^C ages that agree, within the 95% confidence interval, with each other (Table 1), providing support for the local reservoir correction used (140 ± 60 years, Supplementary Note 1). Two ^14^C dates from 564.5 cm (Table 1) were excluded from the age model (Supplementary Note 2); we treat the ages of this section of 117Q (> 10,775 yrs BP) with caution. Sedimentation rates ranged between 0.17 and 0.09 cm yr^−1^ in 117Q BC and the top sections of 117Q and these overlapping core sections span the period − 62 to 372 yrs BP (Fig. 3). Sedimentation rates in 117Q vary from 0.03 to 0.04 cm yr^−1^ and 0.07 to 0.08 cm yr^−1^ (Fig. 3).

**Table 1:** Radiocarbon dates and modelled ages for 117Q and CASQ 1. For details on the correction to 117Q bulk organic carbon dates prior to modelling see Supplementary Note 2. All ^14^C ages were calibrated using the Marine13 dataset^70^ and a local reservoir correction (ΔR) of 140 ± 60 years was applied in the age modelling software BACON^68^. Minimum and maximum ages represent the 95% (2σ confidence interval) in the age model. ** ^14^C dates excluded from the age depth model.

Lab code	Depth in core (cm)	Material	^14^C age (yrs)	^14^C (± yrs)	Bulk organic ^14^C age after correction (yrs BP)	Modelled age (yrs BP)		
						Min	Max	Median
**117Q**								
Beta-507517	18.5	Bulk organic carbon	2370	30	199	37	192	92
Beta-507518	68.5	Bulk organic carbon	3560	30	1377	695	1076	854
Beta-507519	98.5	Bulk organic carbon	4310	30	2119	1381	1808	1574
Beta-507520	144.5	Bulk organic carbon	5620	30	3416	2843	3317	3087
ETH-87284.1.1	199.5	Mixed benthic foraminifera	4725	70		4376	4947	4688
UA-56315	240.5	Bivalve shell fragments	5310	30		5330	5725	5537
ETH-87283.1.1	283.5	Benthic foraminifera (mixed species)	6300	60		6183	6660	6451
ETH-87282.1.1	343.5	Benthic foraminifera (mixed species)	6765	60		7033	7442	7237
ETH-87281.1.1	403.5	Benthic foraminifera (mixed species)	7705	70		8043	8454	8259
ETH-87281.3.1	403.5	Benthic foraminifera (mixed species) (duplicate)	8010	70				
UA-56314	480.5	Bivalve shell fragments	9610	40		9895	10,399	10,226
UA-56313	492.5	Bivalve shell fragments	9675	40		10,205	10,557	10,397
ETH-90546.1.1	524.5	Benthic foraminifera (mixed species)	9970	90		10,597	11,016	10,775
ETH-90547.1.1	524.5	Planktonic foraminifera (*N. pachyderma* sin.)	9875	90				
ETH-90548.1.1	524.5	Bivalve shell fragments	9800	90				
ETH-90544.1.1**	564.5	Benthic foraminifera (mostly *C. neoteretis*)	11,585	90				
ETH-90545.1.1**	564.5	Benthic foraminifera (mixed species)	30,160	290				
**CASQ1**								
ULA-6034	117.5	Bivalve shell fragments	1570	20		729	1018	894
ULA-5837	176.5	Bivalve shell fragments	1850	20		1133	1381	1265
ULA-6035	263.5	Bivalve shell fragments	2370	20		1686	1938	1815
ULA-5836	332.5	Bivalve shell fragments	2660	25		2122	2332	2234
ULA-6036	341.5	Bivalve shell fragments	2705	20		2179	2399	2290
ULA-6037	393.5	Bivalve shell fragments	2970	25		2562	2838	2685
ULA-6044	405.5	Bivalve shell fragments	3505	20		2674	2978	2806
ULA-6045	460.5	Bivalve shell fragments	3505	20		3050	3378	3214
ULA-6046	472.5	Bivalve shell fragments	3775	20		3128	3481	3301
ULA-6047	501.5	Bivalve shell fragments	3485	25		3274	3700	3456
ULA-5835	543.5	Bivalve shell fragments	3745	25		3529	4073	3753

**Figure 4:**
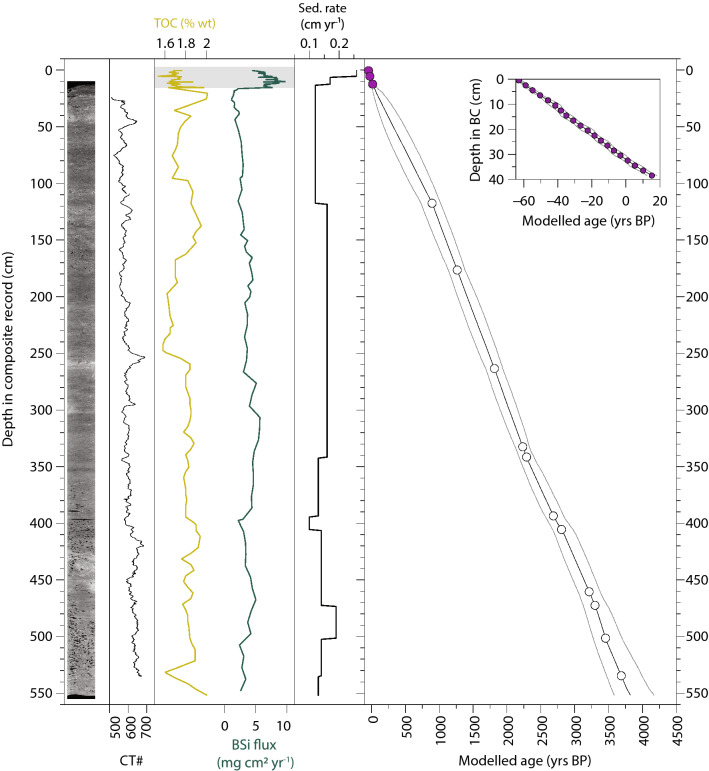
Physical and selected biogeochemical sediment properties and age-depth model for CASQ1 BC and CASQ1. From left to right: CASQ1 Computerised tomography (CT) scan image and CT number (#). Denser areas appear whiter in the CT scan image. Percentage (weight) of total organic carbon (TOC; yellow) and biogenic silica fluxes (BSi; dark green). Calculated sedimentation rates (cm yr^-1^) are shown in black. The median age (modelled) -depth relationship constructed in BACON for CASQ1 and CASQ1 BC (insert) are shown with purple-filled circles representing ^210^Pb-dated intervals and open circles the ^14^C-dated intervals (biogenic carbonate). The solid black line indicates the median modelled age-depth relationship and the grey dashed lines indicate maximum and minimum modelled ages in the 95% confidence interval. . The grey bar indicates the stratigraphic interval where CASQ1 BC and CASQ1 proxies overlap.

The combined ^210^Pb (n = 20) and ^14^C (n = 11) chronology of CASQ1 and CASQ1 BC indicate that the whole core records span the period 3822 (± 291 yrs) to − 43 yr BP (Fig. 4). CASQ1 BC spans 1931–2014 AD with sedimentation rates ranging between 0.39 and 0.63 cm yr^−1^. CASQ1 and CASQ1 BC sediments overlap (top of CASQ1 dated 1993 AD; Supplementary Fig. 2). Sedimentation rates are relatively high and constant throughout most of the CASQ1 sedimentary sequence (between 0.1 and 0.17 cm yr^−1^) (Fig. 4) except near the top of the core (0-33.5 cm) where sedimentation rates peak at 0.6–0.5 cm yr^−1^ .

### Physical, elemental and biogeochemical sedimentary properties

The base of 117Q (586–565 cm; ~ 11,980–11,650 yrs BP) is characterised by laminated mud and CT numbers (or Hounslow units; HU) that primarily reflect bulk densities of ~ 800 HU (Fig. 3). Higher Al + K + Si contents (normalised by the sum of all element counts) indicate a high proportion of locally-sourced terrigenous sediment, likely originating from the clay-rich Inglefield Land (Thule Group)^30^ (Fig. 3). Directly above is a ~ 30 cm thick layer of dense (HU > 850) and coarse (> 63 µm = 18%) sediment composed of large clasts in a somewhat finer matrix, ending abruptly at ~ 10,950 yrs BP. From the bottom and up to ~ 450 cm core depth, the > 63 µm fraction remains low (~ 2%) with the exception of a few larger clasts, found at 560.5, 554–550 and 544–542 cm. From 450 cm core depth (~ 8300 yrs BP), higher Ca content (normalised by the sum of all element counts) suggests a relative increase in Nares Strait-sourced detrital carbonate (e.g.^31^) (Fig. 3). CASQ1 density fluctuates between 500 and 700 HU and despite a lack of grain size measurements, CT images indicate relatively homogenous sediment with no evidence of ice-rafted debris (IRD) (Fig. 4).

Changes in total organic carbon (TOC, % weight) and biogenic silica fluxes (BSi, mg cm^2^ yr^−1^) indicate changes in biological productivity in the NOW region. TOC in 117Q varied between 0.2% at the base of the core to 2% in more recent sediments (Fig. 3). Biogenic silica fluxes (Fig. 3) varied between 0.02 mg cm^2^ yr^−1^ at the bottom and 1.8 mg cm^2^ yr^−1^ in the upper centimetres of the box core. We interpret low BSi fluxes in 117Q with caution; the silica budget (today supplied from Pacific Waters via the Bering Strait) was likely variable through time, particularly before the more direct connection with the Arctic Ocean was established by the opening of the Nares Strait and the establishment of modern circulation (i.e., net outflow of Arctic waters) via the Canadian Arctic Archipelago at ~ 6 kyr BP^32,33^. TOC varied between 1.39 % (~ 200 yrs BP) and 2.01% in CASQ1 and CASQ1 BC (Fig. 4). BSi fluxes vary between 1.2 and 4 mg cm^2^ yr^−1^ in CASQ1 but are significantly higher in CASQ 1 BC, peaking at 9 mg cm^2^ yr^−1^ (Fig. 4).

Total sulphur (TS), measured only in 117Q, is used here as a proxy for deep ventilation/bottom water renewal, as pyrite is more readily deposited in anoxic marine sedimentary settings or where there is insufficient oxygen to oxidise matter infaunally (e.g.^34^). TS varies between 0.09 and 1.02% wt. Values are < 0.4% for large parts of the core but are consistently higher between 8300 and 2800 yrs BP (Fig. 3).

### Foraminifera

**Figure 5:**
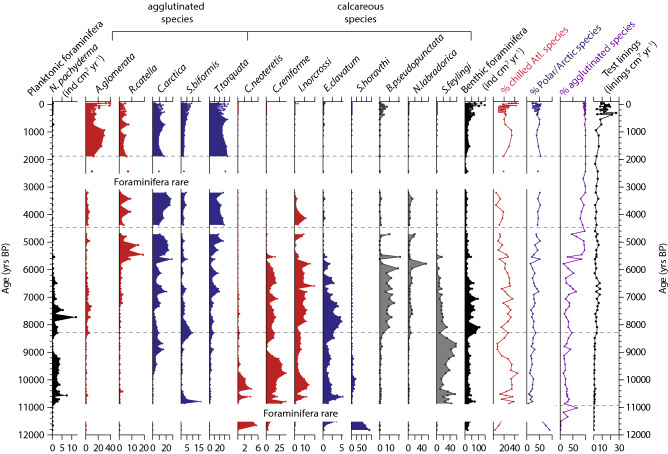
Foraminifera fluxes and assemblages in 117Q and 117Q BC. Planktonic foraminifera (*N. pachyderma* sin.) fluxes are shown in black. Benthic foraminifera assemblages are show as percentage of the entire assemblage and colour coded according to Table 2. Only species that account for > 5% of the total assemblage in at least 1 sample are shown. Total benthic foraminifera fluxes (black) are shown as well as the overall (agglutinated and calcareous) % of chilled Atlantic water (red) and Polar/Arctic (blue) indicator species.

**Figure 6:**
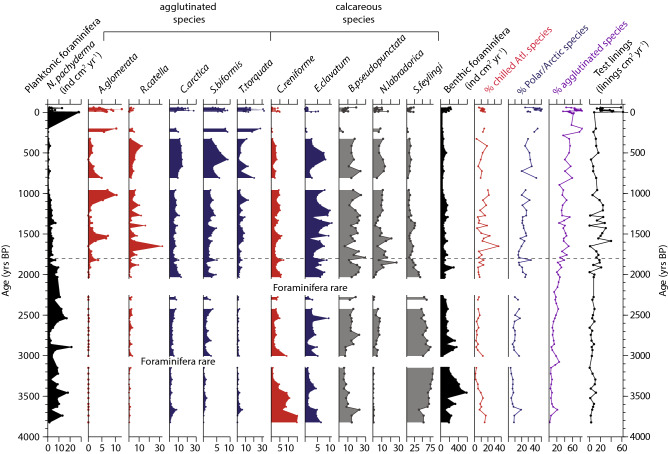
Foraminifera fluxes and assemblages in CASQ1 and CASQ1 BC. Planktonic foraminifera (*N. pachyderma* sin.) fluxes are shown in black. Benthic foraminifera assemblages are show as percentage of the entire assemblage and colour coded according to Table 2. Only species that account for > 5% of the total assemblage in at least 1 sample are shown. Total benthic foraminifera fluxes (black) are shown as well as the overall (agglutinated and calcareous) % of chilled Atlantic water (red) and Polar/Arctic (blue) indicator species.

Benthic foraminiferal fluxes in the two cores ranged from 580 to 0 individuals cm^2^ yr^−1^ and were generally higher in CASQ1 (Figs. 5 and 6). Planktonic foraminifera (*Neogloboquadrina pachyderma* sinistral*)* were absent or rare (< 30 ind cm^2^ yr^−1^) in both cores (Figs. 5 and 6). 47 benthic foraminifera species (20 agglutinated taxa) were identified in the cores (Supplementary Table 1). Variations in the relative abundance of benthic species are used to qualitatively reconstruct water mass characteristics (i.e. temperature and salinity). Based on previous foraminifera studies from the region^28,35–39^, two main groups were defined: the Polar/Arctic water group and the chilled Atlantic water group (Table 2). The Polar/Arctic water group consists of agglutinated cold water species such as *Cuneata arctica* and *Textularia torquata* and the calcareous species *Elphidium clavatum*. The Atlantic water group consists of the calcareous species *Cassidulina reniforme* and *Islandiella norcrossi,* both found in glaciomarine environments under the influence of (chilled) Atlantic-sourced waters along the West Greenland coast^35,36,38,39^ and the agglutinated species *Adercotryma glomerata* and *Reophax catella. Cassidulina neoteretis* is seen as an indicator of a true (warm) Atlantic water influence^37,40^, but as they were rare (≤ 6%) in our records are the grouped with the larger (chilled) Atlantic water group.

**Table 2:** List of benthic foraminiferal key species and associations used in this study.

	Species	References
Polar/Arctic water	Agglutinated	
	*Cuneata arctica* (Brady 1881)	^36,76^
	*Recurvoides turbinatus* (Brady 1881)	^77^
	*Spiroplectammina biformis* (Parker & Jones, 1865)	^76,78^
	*Textularia torquata* (Parker, 1952)	^79^
	Calcareous	
	*Elphidium clavatum* Cushman, 1930	^78,80^
	*Islandiella helenae* Feyling-Hanssen & Buzas, 1976	^81^
	*Stainforthia concava* (Höglund, 1947)	^78,82^
	*Stetsonia horvathi* Green, 1959	^43,83^
Chilled Atlantic water	Agglutinated	
	*Adercotryma glomerata* (Brady, 1878)	^36,84^
	*Lagenammina difflugiformis* (Brady, 1879)	^74,76^
	*Reophax catella* (Höglund, 1947)	^85^
	Calcareous	
	*Cassidulina neoteretis* (Seidenkrantz, 1995)	^37,40^
	*Cassidulina reniforme* (Nørvang, 1945)	^80,86^
	*Islandiella norcrossi* (Nørvang, 1945)	^35,36,38^

From 11,900–11,000 yrs BP, 117Q recorded low planktonic (< 1 ind cm^2^ yr^−1^) and benthic (0 to < 38 ind cm^2^ yr^−1^) foraminiferal fluxes (Fig. 5). The benthic assemblage (Fig. 5) was dominated by *Stetsonia horvathi* (53–88%) with small proportions (~ 6%) of *C. neoteretis*. Foraminiferal fluxes increased between ~ 10,900–8300 yrs BP and benthic assemblages were dominated by *Stainforthia feylingi* (up to 57%) and chilled Atlantic indicator species (*C. reniforme* and *I. norcrossi*), up to 40%. From 8300–4500 yrs BP, planktonic foraminiferal fluxes peaked at 12 ind cm^2^ yr^−1^ (~ 7600 yrs BP) but were otherwise low. Benthic foraminiferal fluxes increased from 8 to 107 ind cm^2^ yr^−1^. *S. feylingi* was replaced by the calcareous species *Nonionella labradorica* (peak at 60%) and *Bolivina pseudopunctata* (15 – 20%) and the chilled Atlantic water group accounted for 11–35%. From 4500–1800 yrs BP, benthic foraminiferal fluxes were low (< 20 ind cm^2^ yr^−1^) and planktonic foraminifera were rare. Polar/Arctic water group species (agglutinated) accounted for ~ 50% and the chilled Atlantic water species group represented only 10% during this interval. From 1800–150 yrs BP benthic foraminiferal fluxes recovered slightly, and assemblages were dominated by the Polar/Arctic indicator *T. torquata* (~ 40%), while the proportion of the chilled Atlantic water indicator *A. glomerata* also increased (~ 20%). In the last 150 yrs, benthic foraminiferal fluxes were ~ 150 ind cm^2^ yr^−1^, with the chilled Atlantic water group representing ca. 40% of the assemblage. Agglutinated taxa (Fig. 5) represented between 16–35% of the total benthic foraminifera assemblage between ~ 11,900 and 8300 yrs BP, with the exception of a peak contribution (70%) ~ 11,000 yrs BP (Fig. 5). From 8300–4500 yrs BP the proportion of agglutinated species increased but was highly variable (25–95%). From 4500 yrs BP to present agglutinated taxa accounted for nearly 100% of the assemblage. Foraminifera test lining fluxes in 117Q and 117Q BC varied between 0–27 linings cm^2^ yr^−1^ and remained low until ~ 600 yrs BP. Linings were largely of planispiral (types II and IV) and trochospiral (type I) forms^41^.

In CASQ1, planktonic foraminiferal fluxes were low (< 2–12 ind cm^2^ yr^−1^) from ~ 3800–2000 yrs BP with the exception of a peak at 2900 yrs BP (Fig. 6). Benthic foraminiferal fluxes were variable (4–> 150 ind cm^2^ yr^−1^) and assemblages were dominated by *S. feylingi* (47–75%) (Fig. 6). Despite the presence of some chilled Atlantic water species (*C. reniforme* = 15%) and Polar/Arctic species (*E. clavatum* = 7.5%), the overall proportion of indicator groups remained low (< 20%). From 2500–2000 yrs BP both planktonic and benthic foraminiferal fluxes were too low for assemblage counts. After 2000 yrs BP, proportions of *B. pseudopunctata* (up to 20%) and *N. labradorica* (~ 15%) increased. The appearance of *A. glomerata* (< 10%) and *R. catella* (up to 20%) at ~ 2000 yrs BP accounts for an increase in the chilled Atlantic water group. From 800 to 200 yrs BP, there was little variation in assemblage composition. In the last ~ 60 yrs, benthic foraminiferal fluxes increased (~ 200 ind cm^2^ yr^−1^) and the Polar/Arctic water group species (agglutinated) became increasingly dominant (50%). The calcareous species *N. labradorica* and *B. pseudopunctata* were present in relatively low abundances (~ 5 to 15%). The proportion of agglutinated species in CASQ1 was between 10–30% from 3800 to 1800 yrs BP and 40–85% after 1800 yrs BP (Fig. 6). Foraminifera test lining fluxes in CASQ1 and CASQ1 BC varied between 0–40 linings cm^2^ yr^−1^ and were mostly of planispiral (types II and IV) and trochospiral (type I) forms^41^. Foraminifera test linings fluxes increased between ~ 2000–1000 yrs BP and during the last ~ 50 years.

### Lipid biomarkers

**Figure 7:**
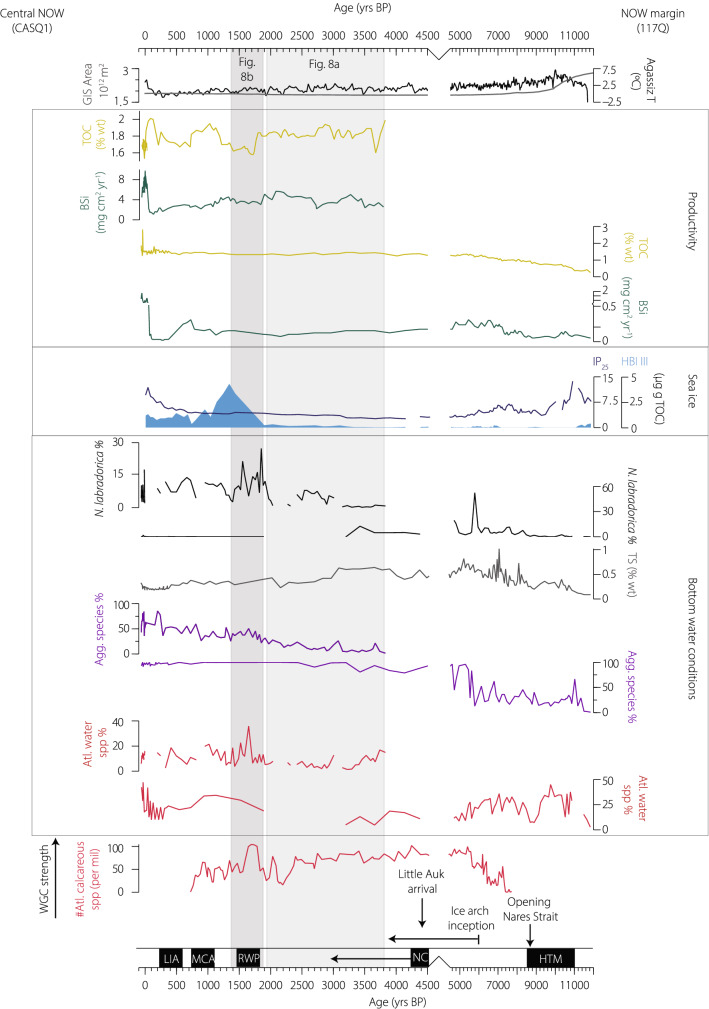
Polynya dynamics and interactions with bottom water conditions in the NOW region during the Holocene. Top to bottom: Modelled Greenland Ice Sheet (GIS) volume^75^ and temperature data from the Agassiz ice core^47^. Data from this study with CASQ1 data plotted on the left y-axis and 117Q on the right y-axis. Total organic carbon % (yellow) and biogenic silica fluxes (dark green) as a measure of biological productivity in the NOW region. The total sulphur (% weight) record for 117Q is indicative of water column stratification and bottom water ventilation state (grey). The proportion of the benthic foraminifera sea-ice related productivity species *N. labradorica* (black), proportion of agglutinated foraminifera taxa (purple) and proportion of chilled Atlantic group species (red) are shown for both cores. WGC strength based on abundances of Atlantic water indicator species within benthic foraminifera assemblages in Disko Bugt^38^ are shown for comparison. Major climatic intervals and shown in black boxes; *HTM* Holocene Thermal Maximum, *NC* Neoglacial cooling, *RWP* Roman Warm Period, *MCA* Medieval Climate Anomaly, *LIA* Little Ice Age. Major changes from regional NOW records area indicated with arrows^16–18,29^. Grey bands highlight periods of inferred strong and weak polynya settings illustrated in Fig. 8. Note the expanded X-axis from 4500 yrs BP–present.

In 117Q, IP_25_ concentrations normalised to TOC ranged from 2.25–13.65 µg g TOC^−1^, peaking between 550–510 cm (~ 11,177–9965 yrs BP), and increased again in the top 30 cm of the core (last ca. 232 yrs BP) (Fig. 7). HBI III (triene) was either absent or present in low concentrations (≤ 0.33 µg g TOC^−1^) in the lower sections of 117Q, and concentrations were consistently higher (> 0.33–4.3 µg g TOC^−1^) in the top 100 cm of the core, with a sharp increase after ca. 1800 yrs (Fig. 7).

## Discussion

Recent work has highlighted changes in the NOW region during the Holocene, both in terms of the variability of ice arch formation that provides the physical preconditioning for NOW formation^17^ and the impact of NOW variability on little auk colonies^16^. We address a further dimension of the polynya system; the previously unassessed relationship between polynya dynamics and bottom water conditions in northernmost Baffin Bay. Our multi-proxy reconstructions from both the eastern peripheral margin and central polynya sites indicate that the physical processes resulting from a highly productive and strong NOW exerted significant influence on bottom ocean conditions from ~ 4400 kyr BP. During warm periods in the late Holocene (particularly the Roman Warm Period), a weak NOW was coeval with increased inflow of Atlantic-sourced waters, testifying to the reduced capacity of the polynya to influence bottom ocean conditions during warmer climatic intervals.

The very cold surface-ocean and harsh sea-ice conditions captured in the bottom part of 117Q likely correspond to the last ~ 200 years of the Younger Dryas stadial (11,900–11,700 yrs BP). Laminated sediments with low sand contents and an absence of IRD reflect the site’s proximity to an ice-sheet margin and subglacial sedimentary input sourced locally; higher proportions of clay minerals (Al, K and Si; Fig. 3) suggest this terrigenous input originated from the Inglefield Bredning region^30^, where the retreat of the Greenland Ice Sheet (GIS) toward its present-day position occurred at 11,900 (± 600) yrs BP^42^. Increasing IP_25_ concentrations and the dominance of *S. horvathi* (Fig. 5), a benthic foraminifera species often found in permanently sea-ice covered areas of the high latitudes with low phytodetritus accumulation^43^, further advocates for an ice-sheet marginal setting. The presence of *C. neoteretis* in our record, nearby cores^28^ and as far north as Smith Sound^19^ indicates chilled Atlantic water influence at this time. A coarse sediment layer in the latter part of this interval (~ 11,500–11,000 yrs BP; Fig. 3) reflects either local deposition (e.g., iceberg rafting) or a mass transport event owing to fast retreat of glacial ice margins. Despite chronological uncertainties for the early Holocene interval, our data indicate extensive sea-ice cover at site 117Q and strong northward penetration of Atlantic-sourced waters via the WGC (Fig. 7).

The influence of Atlantic water persisted post-11,000 yrs BP at the eastern NOW site and this is consistent with increased WGC/EGC inflow observed at more southerly locations along the West Greenland margin from ~ 11,000 yrs BP^44^. The dominance of *S. feylingi* suggests the presence of a nearby sea-ice edge^45^ as well as a high productivity and low oxygen environment^28^, the latter supported by increasing sulphur precipitation (TS) during the early part of this interval (Fig. 3). As the GIS retreated toward the mouth of Inglefield Fjord^46^ during what is considered the Holocene Thermal Maximum (HTM) in this region^47^, temperatures 2.5–3 °C higher than present were recorded in the nearby Deltasø lake from ~ 10,000 yrs BP^48^. Coupled with continued Atlantic water influence seen in our record and others in the region^28^, both factors likely played a role in local ice-stream retreat and species that represent this chilled Atlantic water group (*C. reniforme* and *I. norcrossi*; Fig. 5) are also indicative of glaciomarine conditions^36^. Major reconfigurations of the GIS and Innuitian Ice Sheet (IIS) in northern Baffin Bay during the early- to mid-Holocene culminated in the opening of the Nares Strait between 9000 and 8300 yrs BP^18,19,29^. The subsequent collapse of the ice saddle in Nares Strait is thought to have increased glacial fluxes^49^ and we note a relative increase in detrital carbonate (Ca) in our records at this time, coeval with decreasing local terrigenous input from Inglefield Fjord (Al + K + Si) (Fig. 3). Despite the warmer atmospheric conditions and continued northward ocean heat transport via the WGC, biological productivity remained low and IP_25_ fluxes indicate presence of seasonal sea-ice (Fig. 7). Together, our proxy data points toward an ice-sheet proximal setting between ~ 11,000–8300 yrs BP, with no apparent polynya formation, but continual influence of an extensive WGC.

The resulting physical (and oceanic) setting of northernmost Baffin Bay following the opening of Nares Strait may, in principle, have allowed for polynya formation from ~ 8300 yrs BP. Our central NOW record does not cover this period, but sea-ice-free conditions for 4–5 months per year were inferred from core 91-039-008P nearby our central site^50^. High diatom productivity from ~ 7400 yrs BP was seen as evidence of polynya conditions off Jones Sound^27^ and there was an increase in the relative abundance of phototrophic dinoflagellate cyst species at the outlet of Lancaster Sound^51^. An increase in biological productivity, expressed by increasing BSi fluxes, is evident at our peripheral NOW site during this interval. However, high (but variable) proportions of chilled Atlantic water species suggest a continued influence of the WGC and AMW (Fig. 7) and, coupled with increased precipitation of sulphur (Fig. 3), a well-stratified water column with poorly ventilated bottom waters until ~ 6600 yrs BP—arguing against recurrent polynya formation at this time.

From ~ 6600 yrs BP, increases in biological productivity (BSi) are coeval with a shift toward weaker water column stratification and diminishing influence of chilled AMW (Fig. 7). Conversely, enhanced advection of warm subsurface water onto the east Greenland shelf^37^  likely lead to a strengthening of the WGC in Disko Bay^38^ and Upernavik^39^ around this time (Fig. 7). The rapid increase in the relative abundance of agglutinated taxa at our peripheral NOW site likely reflects the onset of moderate production of corrosive CO_2_-rich brines^23–26^. A similar shift toward agglutinated assemblages ~ 6500 yrs BP and increasing diatom abundance in the western sector of the NOW were attributed to summer open water polynya production^27^. The appearance of the benthic foraminifera species *N. labradorica* (Fig. 7) suggests fresh supplies of phytodetritus from enhanced sea-ice related productivity in the area (e.g.^37^).

These conditions mark the transition from an ice-sheet marginal setting toward a sea-ice marginal setting and the potential presence of an unstable polynya margin. The weakening Atlantic water influence at our site (*vis-à- vis* WGC strengthening further south) could represent influence exerted by the NOW region itself from ~ 6600 yrs BP. We note that the timing of this transitional period is remarkably similar to that in Storfjorden, Svalbard, where higher proportions of agglutinated taxa (and thus increased polynya activity) were evident between ~ 8200 and 4000 yrs BP^24,25^.

From ~ 4400 yrs BP, a major regime shift evident in the wider northern Baffin Bay region marks the inception of stable NOW formation. Lacustrine sediments record the first arrival of little auk colonies, a typical polynya bird^16^. Physical preconditioning for strong NOW formation, namely the harsh sea-ice conditions and the inception of ice arches in Smith Sound, had already begun by ~ 5000 yrs BP^17^, consistent with and likely sustained by a negative phase of the Arctic Oscillation and the regional Neoglacial cooling starting at ~ 4500 yrs BP^17,52,53^. At our peripheral NOW site, a decrease in biological production and significant change in bottom ocean conditions is evident; a combination of minimal Atlantic water influence, dominance of agglutinated foraminifera (> 85%) and decreased sulphur precipitation are consistent with a cold and well-ventilated water column (Fig. 7), also observed after ~ 4000 yrs BP at the western margin of the polynya^27^. Benthic foraminifera species within this assemblage such as *C. arctica* and *T. torquata* are typical of Polar/Arctic environments (Table 2). Similar benthic foraminifera assemblages in nearby core 91-039-012P were attributed to inflow of Arctic waters^28^. However, circulation in the NOW region argues against a specific Arctic water mass signal at depth in the eastern NOW^12^, rather the presence of Polynya Winter Water (PWW; Fig. 2).

**Figure 8:**
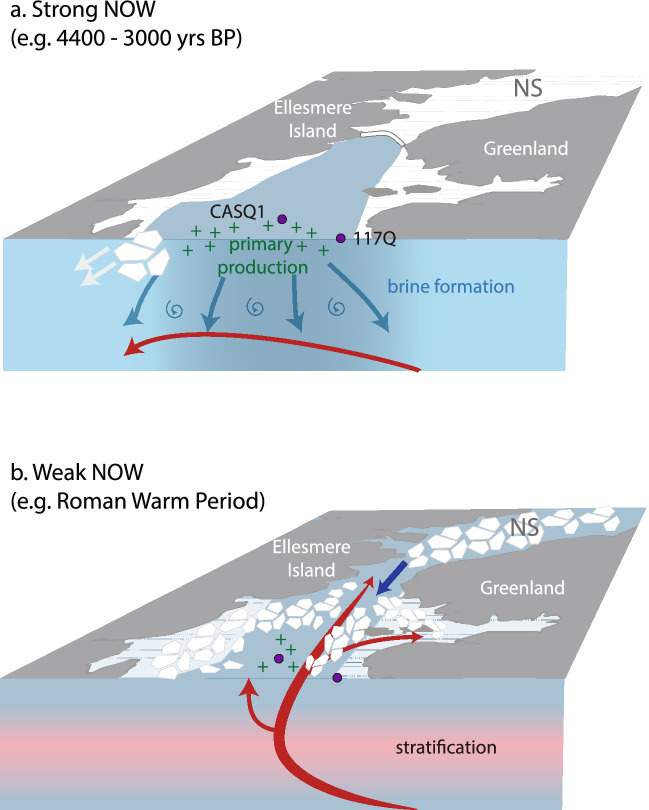
Schematic illustration of contrasting conditions and oceanic configuration in northernmost Baffin Bay/Nares Strait (NS). (**a**) Strong NOW and stable margin; a stable ice arch in Nares Strait facilitates productivity in the large open water area, allowing for the formation of PWW and mixing of brine enriched waters as a result of extensive sea-ice production and subsequent removal southward. This in turn ventilates the water column and diverts the flow of the WGC (red arrows) southward. (**b**) Weak NOW; ice arch instability allows for southward flow of Arctic sea-ice into the NOW region. Productivity is lower in the smaller open water region and weaker brine formation facilities northward penetration of WGC (AMW) into the region, prompting stratification. Purple circles indicate central and peripheral core locations.

The consolidation of an ice arch in southern Kane Basin would not only prevent transport of Arctic sea ice into northernmost Baffin Bay via Nares Strait but also result in mobile, thin new sea-ice conditions south of it, susceptible to export by prevailing winds and hence contributing to open water conditions in the NOW. Repeated cycles of sea-ice formation and removal would have resulted in extensive production of CO_2_-rich, corrosive brines and Polynya Winter Water that would sink and spread, extending down throughout the water column (Fig. 8); a similar structure to that observed during the Godthaab 1928 expedition^21^ (Fig. 2). The overflow of these centrally-generated brines was likely the cause of the dominance of taxa indicative of polar water in the benthic agglutinated foraminifera assemblages^24–26^.

The CASQ1 record, from what is today the central NOW region (Fig. 1), begins at ~ 3800 yrs BP and represents different conditions to those at the peripheral NOW site. Organic carbon and biogenic silica fluxes are an order of magnitude higher in the central NOW (Fig. 7). In the bottom waters of the central NOW, Atlantic water influence is negligible at the seabed and consistent with present and historical hydrography measurements (Fig. 2). Calcareous taxa dominate benthic foraminifera assemblages and as in earlier intervals in the eastern periphery of the NOW, a low oxygen but high productivity benthic environment is inferred by the dominance of *S. feylingi* both in our record (Fig. 6) and nearby core 91-039-008P^28^, likely fuelled by extensive biological productivity and export of organic matter to the seafloor.

Combined with regional records, our data suggest that the NOW exerted a major influence on the northward penetration of chilled Atlantic water  via the WGC from ~ 4000 yrs BP. Despite evidence of an intensified WGC further south in Baffin Bay^38,39^ (Fig. 7), the lateral and vertical extensions of brines generated in the NOW appear to have diverted the flow of the WGC on a more southerly path as flowed east to west across northern Baffin Bay (Fig. 8a). This diversion may have begun already by ~ 6000 yrs BP, when a strong Atlantic water signal was observed for the first time during the Holocene southwest of the NOW region in Lancaster Sound^54^, an influence that persisted throughout the interval of strong NOW formation.

A significant change in sea-surface conditions, biological productivity and bottom water conditions in the NOW region is evident from ~ 1800 yrs BP, concomitant with the onset of the Roman Warm Period (RWP). A sudden and substantial increase of the marginal ice zone (MIZ) biomarker HBI III in our peripheral NOW record indicates a cooling and freshening of surface waters (Fig. 7). Lower biological productivity in the central NOW (Fig. 7) may have been the responsible for low planktonic and benthic foraminifera fluxes and the previously dominant benthic foraminifera species *S. feylingi* is replaced by *B. pseudopunctata* and *N. labradorica*, hinting at sporadic pulses of sea-ice, rather than open water, -related productivity in the central NOW (Figs. 6 and 7). Increasing incursions of Atlantic water at both the central and peripheral NOW sites are most evident during the Rowan Warm Period (RWP) and to a lesser extent the Medieval Climate Anomaly (MCA) (Fig. 7). An increase in subpolar gyre strength and heat advection into the North Atlantic (e.g.^55^) during the RWP was evident as both an increasing Irminger Current component of the EGC in South East Greenland^37^ and strengthened WGC^38^ (Fig. 7).

The chilled Atlantic water signal in the benthic foraminifera assemblages during this time interval is constituted almost entirely of agglutinated taxa (Figs. 5 and 6) and it is important to note that there are only a few studies addressing the sensitivity of these species to environmental conditions (c.f. calcareous taxa) in northernmost Baffin Bay or the Canadian Arctic Archipelago^32,56–58^. Furthermore, higher proportions of agglutinated foraminifera in both cores may seem somewhat counter-intuitive to our inference of a weak NOW and reduced brine production^24–26^. In the central NOW, the shift to chilled Atlantic (agglutinated) taxa is coeval with increasing fluxes of organic test linings, suggesting post-mortem dissolution of calcareous taxa and/or poor preservation of some agglutinated taxa (Fig. 6). Considering our evidence of a weaker NOW and ice-arch instability^17^ during the RWP interval, local sea-ice production and brine formation is unlikely to have been the cause of such corrosive bottom waters. Instead, this points to a highly stratified water column; the advection of saline Atlantic water (at intermediate depth) preventing deeper mixing and leading to a poorly ventilated sediment/water interface bathed in dense, cold waters that would promote biogenic carbonate dissolution. Unlike mid-Holocene increases in WGC strength that were not reflected in our records during the period of strong NOW formation, we attribute the increasing influence of Atlantic water in the NOW region from ~ 1800 yrs BP to reflect the reduced capacity of a weakened NOW to mediate local ocean circulation during warm climatic intervals (Fig. 8b).

During the latter part of the Little Ice Age (LIA), increases in sea ice (IP_25_) are recorded at the peripheral polynya site, suggesting the potential presence of a stable ice arch. Despite a slight decrease in the proportion of benthic Atlantic water species, there appears to be no strong imprint of NOW recovery on bottom ocean conditions. Furthermore, biological productivity did not recover to levels recorded during the previous period of stable NOW formation, despite regional records indicating the return of (smaller) little auk colonies^16^. Over the last ~ 150 years however, increasing biological productivity at the central NOW site, as well as decreasing Atlantic water influence, indicate a somewhat strong NOW. Conversely, at the margins of the NOW, Atlantic water influence and stratification persisted, and enhanced seasonal sea-ice cover is inferred from increasing IP_25_ fluxes (Fig. 7). Organic matter^59^ and dinoflagellate cyst concentrations^8^ were more variable in the polynya periphery, suggesting that its margins were dynamic and productivity was not spatially homogenous (e.g.^7^). Shorter ice-arch duration or even failure^3,60,61^ and positive sea-ice anomalies in northernmost Baffin Bay^3^ hint at the increasing NOW instability in the last decades.

## Conclusions

Using two well-dated marine sediment cores from both the central and eastern periphery of the NOW, we applied a suite of proxies to track the interaction between NOW dynamics and bottom ocean conditions throughout the Holocene. We shift focus from NOW configuration as simply a product of oceanic and atmospheric forcing, to reconcile the impact of, and interactions between, NOW formation and bottom ocean conditions in northernmost Baffin Bay. Our results demonstrate an ice-marginal setting with strong Atlantic water influence in northernmost Baffin Bay during the early Holocene. Following this, the opening of the Nares Strait (~ 9000–8300 yrs BP) potentially prompted the formation of an (unstable) polynya margin, coincident with a weaker influence of Atlantic water in the NOW region . The inception of a stable NOW, congruent with the onset of Neoglacial cooling from ~ 4400 yrs BP, had a clear impact on bottom ocean conditions. Active local sea-ice formation and rapid removal resulted in extensive brine production and hindered the incursion of northward flowing Atlantic water into the region. At ~ 1800 yrs BP, during the Roman Warm Period, a weakened NOW facilitated the northward penetration of Atlantic Water. We highlight a new dimension of the polynya system; the influence that NOW formation has on bottom water conditions and circulation in northernmost Baffin Bay. Declining multiyear Arctic sea-ice^62^ and growing instability in the Nares Strait ice-arch location and duration^14,15,61^ may result in weaker polynya activity and penetration of Atlantic-derived water masses and thus ocean heat further north, potentially resulting in increased melt of the marine-terminating glaciers that flank the coast of northwest Greenland^63^.

## Methods

### Sediment core material

The Calypso Square giant gravity core CASQ1 (77°15.035′ N, 74°25.500′ W, 692 m water depth) and its accompanying box core(s) (BC; same location) were retrieved from Smith Sound aboard the *CCGS Amundsen* ArcticNet 2015 Leg 4a expedition. Total sediment recovery was 543 cm for CASQ1 and 44.5 cm for CASQ1 BC. CASQ 117Q and box cores (77°00.29′ N, 72°08.32′ W, 963 m water depth) were retrieved from outside the Inglefield Bredning Fjord (Qaanaaq) aboard the *CCGS Amundsen* GreenEdge/ArcticNet 2016 Leg 1a expedition. Total sediment recovery was 599 cm for 117Q and 40.5 cm for 117Q BC. U-channels were taken from the CASQ cores and sub-sampled at 1 cm intervals. Push cores were extracted from the box cores during both expeditions split lengthways and sub-sampled at 1 cm. The U-channels were kept cold (2–6 °C) and in the dark. Subsamples for foraminifera and biogeochemical analyses were stored at − 20 °C.

### Geochronology

^210^Pb dating was performed on both box cores and the uppermost samples of the long cores to ascertain continuity and /or overlap between the records. In 117Q BC, a total of 15 ^210^Pb measurements were performed at 1 cm intervals (0.5–9.5 cm 117Q BC core depth) and 2 cm intervals for depths 9.5–19.5 cm (Fig. 3). The top 12.5 cm of 117Q were analysed at 1***–***4 cm depth (Fig. 3, Supplementary Fig. 1). ^210^Pb dating was performed at 2 cm intervals on CASQ1 BC (Fig. 4, Supplementary Fig. 2) and on three samples in the upper 12.5 cm of CASQ1 on an adjacent U-channel (Fig. 4). Samples were analyzed for the activity of ^210^Pb, ^226^Ra and ^137^Cs via gamma spectrometry at the Gamma Dating Center, Department of Geosciences and Natural Resource Management, University of Copenhagen. Measurements were carried out on Canberra ultralow-background Ge-detectors. ^210^Pb was measured via its gamma-peak at 46.5 keV, ^226^Ra via the granddaughter ^214^Pb (peaks at 295 and 352 keV) and ^137^Cs via its peak at 661 keV. The content of unsupported ^210^Pb in 117Q BC showed an exponential decline with depth (Supplementary Figs. 1 and 2). The ^210^Pb-based chronology was calculated using a modified CRS-model^64^; where the activity below the lowermost analyzed sample (39 cm) was calculated on the basis of a regression of unsupported ^210^Pb vs cumulative mass depth in the depth interval 20–39 cm.

### Radiocarbon dating

A total of 28 radiocarbon dates were obtained from both cores. For 117Q, a total of 17 radiocarbon dates were obtained (Table 1); 13 were performed on biogenic carbonate samples of either mixed benthic foraminifera, planktonic foraminifera (*Neogloboquadrina pachyderma* sin.) or bivalve shell fragments picked from dried sediment samples (> 150 µm). The selection of sample material depended upon the availability of sufficient material within the 1 cm interval investigated. Mixed benthic foraminifera samples included the species *C. reniforme*, *E. clavatum* and *N. labradorica* and *C. lobatulus* and one of the dates at 564.5 cm depth was based solely on *C. neoteretis*. Traditional Accelerator Mass Spectrometry (AMS) ^14^C dating was used to date bivalve shells at 3 intervals (Uppsala University, Sweden). For biogenic carbonate dates on foraminifera (n = 10), a new method where ultra-small amounts (~ 0.5 mg) of carbonate^65,66^ were directly analysed in a compact AMS facility equipped with a gas ion source at the Laboratory for Ion Beam Physics, ETH Zurich. Due to the paucity of calcareous foraminifera and shells in the top ca. 200 cm of 117Q, bulk organic carbon dates (n = 4) were additionally obtained via traditional AMS ^14^C dating on dried bulk samples (Table 1). The four bulk organic carbon measurements were corrected using the method proposed by Andrews et al.^67^ (Supplementary Note 2). All radiocarbon dating on CASQ1 was performed on bivalve shells (n = 11, Table 1) using AMS ^14^C dating at the Keck Carbon Cycle AMS Facility, University of California, Irvine, US.

### Age-depth modelling

The mixed age-depth model, using both ^210^Pb and ^14^C dates for both long and short (box) cores, was constructed using BACON^68^, an open-source package of ‘R’^69^. All ^14^C dates and their associated errors were calibrated within the age-depth modelling process using the Marine13 radiocarbon calibration curve^70^ and the additional local reservoir correction (ΔR) of 140 ± 60 years. A full discussion on the selection of an appropriate local reservoir correction is provided in Supplementary Note 1.

### Physical, elemental and biogeochemical sedimentary properties

U-channels were run through a computerized axial tomography scanner (Siemens SOMATOM Definition AS + 128) at the Institut National de Recherche Scientifique (INRS-ETE, Quebec, Canada). Digital X-ray images were used to identify different sedimentary structures, and expressed as computed tomography (CT) numbers to visualise changes in bulk density^71^. High-resolution (0.5 cm) X-Ray Fluorescence (XRF) scanning was conducted on 117Q using an AVAATECH XRF core-scanner as a first order estimation of sediment provenance. Measurements were acquired with generator settings of 10, 30 and 50 kV in order to detect elements in the range of Al to Ba. We determined the contribution of locally-sourced sediments, originating from the clay-rich Inglefield Land (Thule Group)^30^ using the sum of aluminium, potassium and silica (Al, K and Si). A second sediment source of interest was the calcium (Ca) component, representing detrital carbonate sourced from the Palaeozoic limestones and dolostones of Nares Strait region (e.g.^31^). The locally sourced (Al, K and Si) and secondary sourced (Ca) elemental counts were normalised by the sum of all elemental counts per second (Fig. 3).

Total organic carbon (TOC) measurements were carried out at the Geological Survey of Denmark and Greenland. TOC was measured at 5 cm intervals in CASQ1 and 4–8 cm intervals in 117Q. Both box cores were measured at 2 cm intervals. Dried sediment samples (~ 0.5 g) were powdered (< 250 μm) and subjected to Rock–Eval type bulk flow pyrolysis using a HAWK instrument (Wildcat Technologies, Texas). Sets of one control-standard (in-house standard) and one blank were run for every 10 samples in order to ensure instrument stability. In core 117Q and 117Q BC, Total Carbon (TC, weight %), Total Organic Carbon (TOC, weight %) and Total Sulphur (TS, weight %) were determined by combustion in a LECO CS-200 induction furnace. TOC was determined after elimination of carbonate-bonded carbon by prolonged HCl treatment.

Biogenic Silica (BSi) analyses were performed at 1 cm intervals in 117Q BC and 2 cm intervals in CASQ1 BC. A total of 86 samples (1–10 cm intervals) and 80 samples (4–8 cm intervals) were measured from the CASQ1 and 117Q, respectively. BSi concentrations were determined from freeze-dried, manually ground sediment samples using an alkaline extraction with mineral correction. Samples (30 ± 2 mg dry sediment) were extracted in 40 ml of 1% Na_2_CO_3_-solution in a water bath at 85 °C for 5 h. Subsamples of 1 ml were withdrawn from the solution at 3, 4 and 5 h and neutralized with 9 ml of 0.021 N HCl. The concentration of dissolved Si in each subsample was analysed manually by the blue ammonium molybdate method^72^ using a spectrophotometer (Perkin Elmer lambda 25UV/VIS). The BSi concentration of the analyzed sediment was calculated from the intercept of the linear regression equation obtained by plotting the increase in Si against time. The method is based on the assumption that all biogenic Si has dissolved after two hours of extraction, while mineral Si dissolves continuously at a constant rate. BSi data are presented as fluxes to account for down-core changes in sedimentation rate as well as the different temporal resolutions of 117Q and CASQ1. Fluxes were calculated using the mass accumulation rate (MAR, g cm^2^ yr^−1^), calculated from sedimentation rate (LSR) × dry bulk density.

### Lipid biomarkers

Sediment samples for biomarker (IP_25_ and HBI III) analyses were processed following the protocol described by^73^ at Université Laval. An internal standard (7-hexylnonadecane) was added to freeze-dried sediment before treatment. Hydrocarbon fractions were analysed using an Agilent 7890 gas chromatograph (GC) fitted with 30 m fused silica Agilent J&C GC columns and coupled to an Agilent 5975C Series mass selective detector. Oven temperatures were programmed as follows: 40–300 °C at 10 °C/min, followed by an isothermal interval at 300 °C for 10 min. The data were collected using ChemStation and analysed using the MassHunter quantification software. IP_25_ was quantified based on the retention time and comparison of mass spectra with authenticated standards. Analyses were done at a 4–8 cm sampling resolution (114 samples analysed). We report concentrations of IP_25_ and HBI III normalised to TOC, in µg g TOC^−1^.

### Foraminifera

Between 5 to 22 g of wet sediment was used for foraminifera analysis. Frozen samples were soaked overnight in deionised water, gently washed over a 63 µm sieve and subsequently stored in a buffering/storage solution of 30% ethanol and 1.5% sodium carbonate. Foraminifera (planktonic and benthic) were counted from the wet residue (> 63 µm) under a stereomicroscope. This preparation method was used to minimise possible loss or fragmentation of fragile and agglutinated specimens^74^. 117Q was analysed at 4–8 cm intervals and CASQ1 at 5–10 cm intervals. Both box cores were counted at 2 cm intervals. A total of 168 samples were counted and benthic species identified. Foraminifera abundances are shown as fluxes (individuals cm^2^ yr^−1^). Benthic foraminifera assemblage data are only shown for samples where at least 300 benthic specimens were found. The flux of organic linings of foraminifera were not included in benthic foraminifera flux calculations.

## Supplementary Information

Supplementary Information.

## Data Availability

All data presented here will be made available online via the open-access PANGEA database (http://www.pangea.de).
