# Supplementary material for: Holocene polynya dynamics and their interaction with oceanic heat transport in northernmost Baffin Bay
Source: Sci Rep. 2021 May 12;11:10095. doi: 10.1038/s41598-021-88517-9 (PMC8114930; doi:10.1038/s41598-021-88517-9)
Supplement: Supplementary file 1 — Supplementary Information. [file 41598_2021_88517_MOESM1_ESM.pdf]

Holocene polynya dynamics and their interaction with oceanic heat transport in northernmost Baffin Bay

Rebecca Jackson[1]*, Anna Bang Kvorning[1,2], Audrey Limoges[3], Eleanor Georgiadis[4,5], Steffen M. Olsen[6], Petra Tallberg[7], Thorbjørn J. Andersen[2], Naja Mikkelsen[1], Jacques Giraudeau[5], Guillaume Massé[8,9], Lukas Wacker[10] and Sofia Ribeiro[1]

[1]Department of Glaciology and Climate, Geological Survey of Denmark and Greenland, Øster Voldgade 10, Copenhagen 1350, Denmark

[2]Department of Geosciences and Natural Resource Management, University of Copenhagen, Øster Voldgade 10, Copenhagen 1350, Denmark

[3] Department of Earth Sciences, University of New Brunswick, 2 Bailey Drive, Fredericton, E3B 5A3, Canada

[4]Department of Geography, Durham University, Lower Mountjoy South Road, Durham, DH1 3LE, UK

[5]Université Bordeaux, CNRS, EPHE, UMR 5805 EPOC, Allée Geoffroy Saint-Hilaire, CS 50023, 33615 Pessac Cedex, France

[6]Danish Meteorological Institute (DMI), Lyngbyvej 100, 2100 Copenhagen, Denmark

[7]Faculty of Biological and Environmental Sciences, P.O. Box 65, 00014 University of Helsinki, Finland

[8]Station Marine De Concarneau, Place De La Croix, 29900 Concarneau, France

[9]Université Laval, CNRS, UM 3376 TAKUVIK, Allée de la Médecine, G1V0A6 Québec, QC, Canada

[10]Ion Beam Physics, Physics, Swiss Federal Institute of Technology in Zurich, Otto-Stern-Weg 5, 8093 Zürich, Switzerland

*corresponding author: rjac@geus.dk

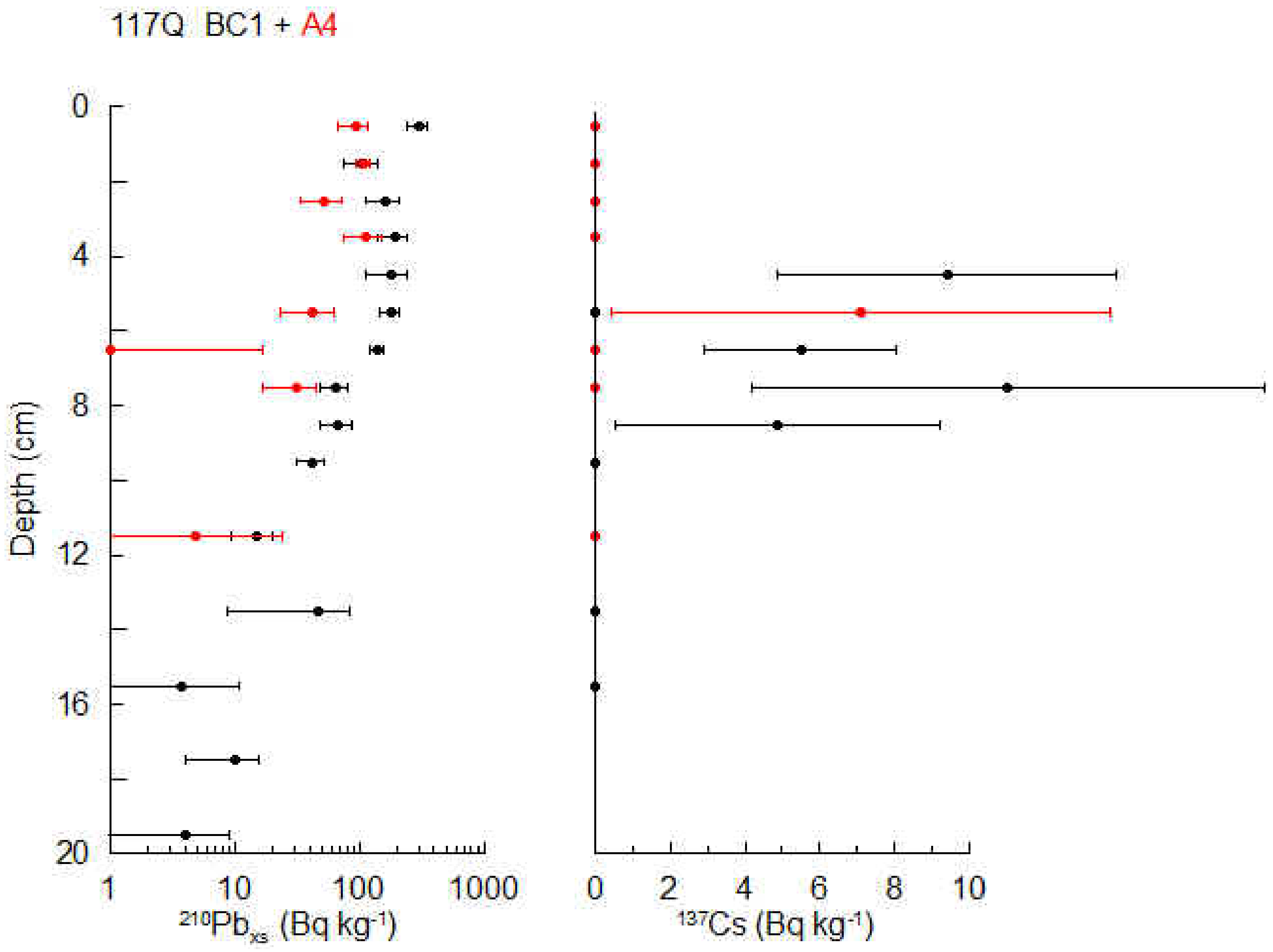

Supplementary figure 1. $^{210}Pb_{xs}$ and ^{137}Cs data for 117Q BC (black) and the top section of 117Q (A4, red), demonstrating a chronological overlap of 6.5 cm between the two sediment cores.

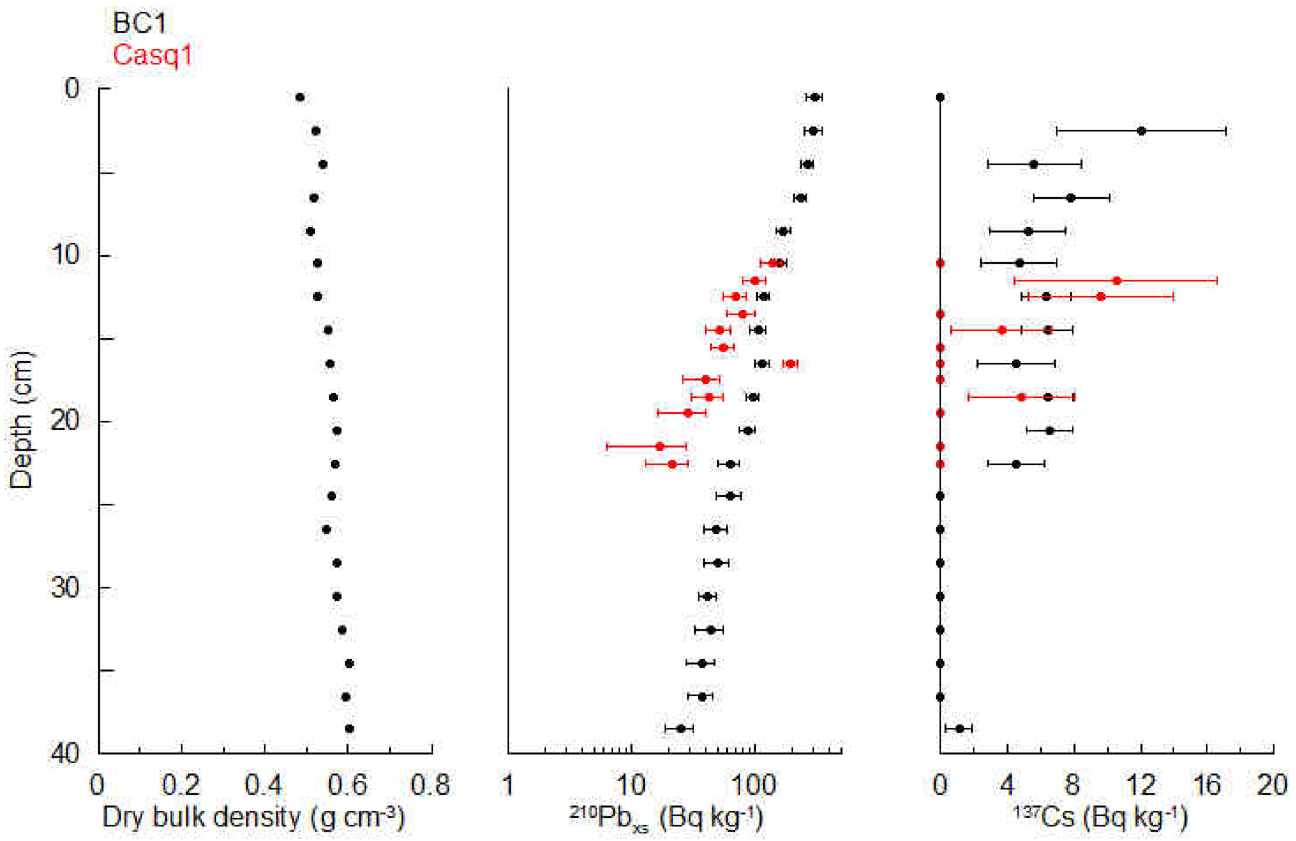

Supplementary Figure 2. Dry bulk density, $^{210}Pb_{xs}$ and ^{137}Cs data for CASQ1 BC (black) and the top section of CASQ1 (red), demonstrating a chronological overlap of 10.5 cm between the two sediment cores.

Supplementary note 1. Reservoir correction (ΔR)

Ages based on ^{14}C measurements from marine sedimentary records require a further local reservoir correction (ΔR) and there are distinct spatial offsets in Arctic regions. Estimates as high as 335 +/- 85 years are proposed for the Canadian Arctic Archipelago[1]. ^{14}C measurements from live marine specimens in Inglefield Bredning fjord (< 40 m water depth) before the mid-1950's indicate a local reservoir correction of -40 years[2]. A deeper site in the NOW region indicates a ΔR of 140 +/- 60 years[2], consistent with offsets calculated in Disko Bay of 140 +/- 35 years[3], an area influenced by the WGC. Furthermore, nearby records from Lancaster Sound employed a Preboreal ΔR of 75 +/- 25 years[4], derived from co-dated cold-water coral records in the north west Atlantic[5]. The water depth and penetration of the WGC at site 117Q means it is unlikely that reservoir corrections are as small (or negative) as those proposed for Inglefield Bredning. Thus, for both 117Q and CASQ1, a local reservoir correction (ΔR) of 140 +/- 60 years (after [2]) is applied to all ^{14}C dates.

To test the appropriateness of the selected reservoir correction, duplicate radiocarbon measurements (Table 1) were carried out at the same 1 cm interval on mixed benthic foraminifera (403.5 cm). Three replicate dates from the same 1 cm interval (524.5 cm) were measured on planktonic foraminifera, mixed benthic foraminifera and bivalve shells respectively. Three replicate ^{14}C dates from the same 1 cm interval (524.5 cm) in 117Q measured on planktonic foraminifera, mixed benthic foraminifera and bivalve shells reported ^{14}C ages that agree, within the 95 % confidence interval, with each other, thus providing support for the local reservoir correction used (140 +/- 60 years) and in the case of the latter interval, no influence of old (or ancient) carbon or reworking. Below 524.5 cm however, two ^{14}C dates on the deepest section of the core (564.5 cm, Table 1) returned high leach fraction values and in the case of the mixed benthic foraminifera date, older than anticipated ^{14}C dates (Table 1). We excluded these two ^{14}C dates from the age-depth model and as such treat the ages of this section (>10,775 yrs BP) with caution.

Supplementary note 2. Bulk carbon ^{14}C dating of 117Q

^{210}Pb dating on 117Q BC and the top section of 117Q confirmed that the cores chronologically overlap by 6.5 cm (Figure S1 and S2). No biogenic carbonate was found between the lowermost ^{210}Pb date (15.5 cm) and uppermost ^{14}C date (199.5 cm). In order to provide age control for this section of the core, four bulk organic samples were radiocarbon dated (Table 1, main text). Bulk dates are often considerably older than their biogenic counterparts in Holocene sediments from Baffin Bay due to the influx of older carbon *via* reworking of marine or terrestrial deposits following the Last Glacial Maximum. When both bulk organic carbon and biogenic carbonate dates (that chronologically overlap) are available, methods such as Tukey resistance line (or median-median line) analysis can be used to statistically correct the age. This has been used in previous studies in Baffin Bay[6], but in the case of 117Q was not possible due to the lack of biogenic carbonate dates. Instead, following the method described by Andrews et al. (1985) using linearly interpolated dates (C) between the lowermost ^{210}Pb date (15.5 cm depth) and the uppermost ^{14}C age (uncalibrated) on mixed benthic foraminifera (199.5 cm) and the bulk organic dates (*O*). The median-median line analysis produced the empirical equation [1]

[1] $O = 1.011C + 2169$

for correcting bulk organic carbon dates (Table 1). After correction, the same marine carbon calibration (Marine13) and local reservoir correction (140 +/- 60 yrs) used for ^{14}C dates on foraminifera shells was applied. The resulting age-depth relationship (Figure 3) indicates that the combined 117Q BC and 117Q records span the period -62 yrs BP (years before present, by conventional radiocarbon dating format; 2012 AD) to 11,984 yrs BP +/- 598 yrs BP.

Supplementary Table 1. Benthic foraminiferal taxa identified in this study

**Foraminiferal taxa**
**Planktonic foraminifera**
Neogloboquadrina pachyderma (sinistral) (Ehrenberg 1861)
**Benthic foraminifera**
Adercotryma glomerata (Brady, 1878)
Ammotium cassis (Parker, 1870)
Astrononion gallowayi Loeblich and Tappan, 1953
Bolivina pseudopunctata Höglund, 1947
Buccella frigida (Cushman, 1922)
Cassidulina neoteretis Seidenkrantz, 1995
Cassidulina reniforme Nørvang, 1945
Cibicides lobatulus (Walker & Jacob, 1798)
Cribrostomoides crassimargo (Norman, 1858)
Cribrostomoides jeffreysi (Williamson, 1958)
Cuneata arctica (Brady, 1881)
Dentalina sp.
Deuterammina sp.
Eggerella advena (Cushman, 1922)
Elphidium excavatum (Terquem) forma clavata Cushman, 1930
Elphidium sp.
Epistominella sp.
Fissurina sp.
Globobulimina auriculata arctica (Höglund, 1954)
Islandiella helenae Feyling-Hanssen and Buzas, 1976
Islandiella norcrossi (Nørvang, 1945)
Lagenammina difflugiformis (Brady, 1879)
Melonis barleeanus (Williamson, 1858)
Nonionella tugida var. digitata Nørvang
Nonionellina auricula Heron-Allen and Earland, 1930
Nonionellina labradorica (Dawson, 1860)
Parafissurina sp.
Portatrochammina bipolaris (Brönnimann & Whittaker, 1980)
Pullenia osloensis Feyling-Hanssen, 1954
Pyrgo williamsoni (Silvestri, 1923)
Quinqueloculina arctica Cushman, 1933
Recurvoides turbinatus (Brady, 1881)
Reophax catella Höglund, 1947
Reophax catenatus Höglund, 1947
Reophax fusiformis (Williamson, 1858)
Reophax pilulifer Bandy, 1884
Reophax scorpiurus Montfort, 1808
Reophax subfusiformis Earland Em. Höglund, 1947
Reophax sp.
Robertina arctica d'Orbigny, 1846
Silicosigmoilina groenlandica (Cushman, 1933)
Spiroplectammina biformis (Parker & Jones, 1865)
Stainforthia concava (Höglund, 1947)
Stainforthia feylingi Knudsen & Seidenkrantz, 1994

Stainforthia sp.
Stetsonia horvathi Green, 1959
Textularia earlandi Phleger, 1952
Textularia torquata Parker, 1952
Triloculina sp.
Trochammina sp.

1. Coulthard, R. D., Furze, M. F. A., Pieńkowski, A. J., Chantel Nixon, F. & England, J. H. New marine ΔR values for Arctic Canada☆. *Quat. Geochronol.* **5**, 419–434 (2010).

2. McNeely, R. & Brennan, J. *Geological Survey of Canada revised shell dates, Open file 5019*. (2005). doi:10.4095/221215

3. Lloyd, J. *et al.* A 100 yr record of ocean temperature control on the stability of Jakobshavn Isbrae, West Greenland. *Geology* **39**, 867–870 (2011).

4. Furze, M. F. A. *et al.* Deglaciation and ice shelf development at the northeast margin of the Laurentide Ice Sheet during the Younger Dryas chronozone. *Boreas* (2017). doi:10.1111/bor.12265

5. Cao, L., Fairbanks, R. G., Mortlock, R. A. & Risk, M. J. Radiocarbon reservoir age of high latitude North Atlantic surface water during the last deglacial. *Quat. Sci. Rev.* **26**, 732–742 (2007).

6. Andrews, J. T., Jull, A. J. T., Donahue, D. J., Short, S. K. & Osterman, L. E. Sedimentation rates in Baffin Island fiord cores from comparative radiocarbon dates. *Can. J. Earth Sci.* **22**, 1827–1834 (1985).
